# Improving the assessment of adverse drug reactions using the Naranjo Algorithm in daily practice: The Japan Adverse Drug Events Study

**DOI:** 10.1002/prp2.373

**Published:** 2018-01-03

**Authors:** Hiroki Murayama, Mio Sakuma, Yuri Takahashi, Takeshi Morimoto

**Affiliations:** ^1^ Department of Clinical Epidemiology Hyogo College of Medicine Nishinomiya Japan

**Keywords:** adverse drug reactions, categorization, daily practice, JADE study, modification, Naranjo Algorithm, patient safety, pharmacovigilance, sensitivity, specificity

## Abstract

It is difficult to determine adverse drug reactions (ADRs) in daily complicated clinical practice in which many kinds of drugs are prescribed. We evaluated how well the Naranjo Algorithm (NA) categorized ADRs among suspected ADRs. The Japan Adverse Drug Events (JADE) study was a prospective cohort study of 3459 inpatients. After all suspected ADRs were reported from research assistants, a single physician reviewer independently assigned an NA score to each. After all NA score of suspected ADRs were scored, two physician reviewers discussed and determined ADRs based on the literature. We investigated the sensitivity and specificity of NA and each component to categorize ADRs among suspected ADRs. A total of 1579 suspected ADRs were reported in 962 patients. Physician reviewers determined 997 ADRs. The percentage of ADRs was 94% if the total NA score reached 5. The modified NA consisted of 5 components that showed high classification abilities; its area under the curve (AUC) was 0.92 for categorizing ADRs, the same as the original. When we set the total NA score cut‐off value to 5, specificity was 0.95 and sensitivity was 0.59. When we reclassified NA components as binary variables, the specificity increased to 0.98 with a cut‐off value of 4 and yielded an AUC of 0.93. In conclusion, we showed that both NA and modified NA could categorize ADRs among suspected ADRs with a high likelihood in daily clinical practice.

**Abbreviations**
ADRs: adverse drug reactions
AUC: area under the curve
JADE: The Japan Adverse Drug Events
NA: Naranjo Algorithm
ROC: receiver operating characteristic

## 1 INTRODUCTION

Discriminating adverse drug events (ADRs) from various symptoms in daily practice is important in order for physicians to take action to mitigate the adverseness and prevent recurrence. However, patients are usually treated with many kinds of drugs, which make it difficult to identify an ADR in daily practice. A tool to categorize ADRs among complicated suspected symptoms could be useful for healthcare professionals to take action proactively as well as to confirm the probability of ADRs retrospectively.

Naranjo et al proposed a tool to evaluate the probability of true ADRs from suspected ADRs,^1^, ^2^ and it has been widely used as the Naranjo Algorithm (NA).^3^, ^4^, ^5^, ^6^ In addition to the NA, several assessment tools have been developed, such as the Liverpool adverse drug reaction causality assessment tool^7^ and the French Causality Assessment Method.^8^ These tools are used to evaluate the probability of an ADR rather than to screen ADRs from suspected ADRs prospectively to take action. While the NA is a traditional tool, it consists of 10 components, and it is complicated to calculate the total score and would require time to utilize it in a daily clinical setting. To save time and resources, a convenient tool to categorize ADRs with high specificity is needed.

We recently conducted the Japan Adverse Drug Events (JADE) study, which evaluated the incidence of ADRs and medication errors among Japanese hospitalized inpatients.^9^, ^10^, ^11^, ^12^, ^13^, ^14^ In the present study, we evaluated the usefulness of the NA to categorize ADRs among suspected ADRs using the JADE database and tried to modify it into a convenient tool to use in daily clinical practice.

## 2 MATERIALS AND METHODS

### 2.1 Study design and patient population

The JADE study was a multicenter prospective cohort study that included 3459 inpatients aged ≥15 years. The study site was three urban tertiary care hospitals in Japan, patients admitted at 15 randomly selected medical and surgical wards as well as three intensive care units from January through June 2004 were eligible for this study.^9^ The institutional review boards of the three participating hospitals approved the study. Informed consent was waived because all data were collected in daily practice.

### 2.2 Naranjo Algorithm

The NA consists of 10 components assessing the likelihood of ADRs.^1^, ^2^ Each component is scored from −1 to +2 based on the findings of each event, including (1) previous conclusive reports, (2) time course, (3) improvement after withdrawal or treatment, (4) re‐emergence after re‐challenge, (5) other causative conditions of symptoms, (6) response to placebo if used, (7) evidence in blood of toxicity, (8) dose response, (9) similar reactions before, and (10) other objective evidence.

### 2.3 Data collection and review process

Research assistants, who were trained nurses or nursing students, reviewed all medical charts, along with laboratory results, incident reports, and prescription queries by pharmacists with the standardized form daily. They reported any suspected ADRs that might be potential ADRs in a standard manner.^15^ After all suspected ADRs were reported from research assistants, a single physician reviewer independently assigned an NA score to each suspected ADR. After all NA score of suspected ADRs were scored, two independent physician reviewers evaluated all suspected ADRs and classified them as confirmed ADRs or not. If discordance happened, such discordance was resolved through discussion to reach consensus.

### 2.4 Statistical analyses

A continuous variable is presented as the mean ± standard deviation (SD) and categorical variables are shown as numbers and percentages. We expressed the distribution of NA scores in each component as the percentage of confirmed ADRs among suspected ADRs for each score in each component. We evaluated the percentage of confirmed ADRs among suspected ADRs for each total NA score. ADRs which are confirmed by physician reviewers are considered as true positive. All suspected ADRs were categorized as positive or negative based on the NA score; then sensitivity and specificity were calculated by these figures. We constructed a receiver operating characteristic (ROC) curve for the summed score of all and selected NA components to compare the categorization abilities of original and modified NA scores. To simplify the NA for convenient use, we reclassified NA components as binary variables. For example, an NA component that had three possible scores, such as +2, 0, and −1 or +1, 0, and 1, were converted to +1 and 0 in which the positive score was converted to +1 and the 0 and negative scores were summarized as 0. We carried out all analyses using JMP 11.2 (SAS Institute Inc., Cary, NC, USA) software.

## 3 RESULTS

**Figure 1:**
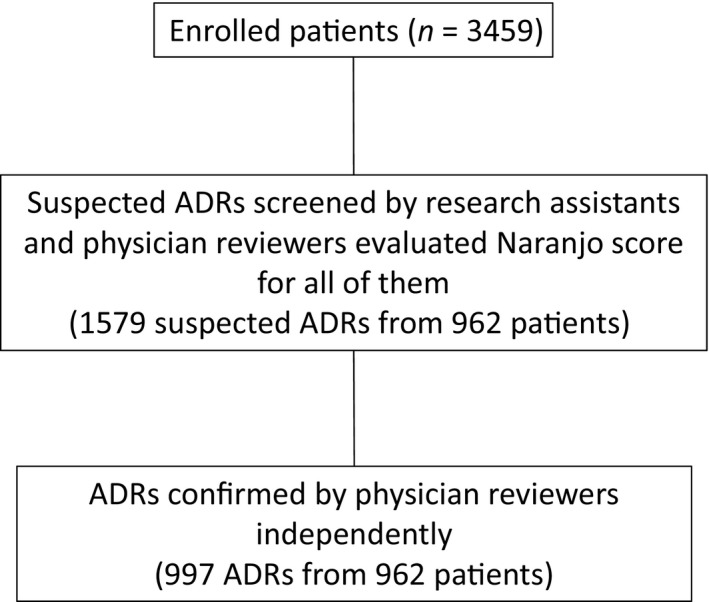
Evaluation process for adverse drug events (ADRs). ADRs were evaluated using 3 steps. Research assistants suggested suspected ADRs from potential drug‐related incidents. A physician reviewer scored each suspected ADR independently using the NA. Two physician reviewers identified ADRs based on consensus of an expert panel

There were 1579 suspected ADRs occurring in 962 patients from among 3459 patients enrolled (Figure 1). Physician reviewers finally concluded that 997 actual ADRs occurred from among the suspected ADRs. Among the 962 patients with NA scores, 517 (54%) were men and the mean age was 70 (SD 15) years. The medical and surgical wards and the ICUs admitted 437 (45%), 410 (43%), and 115 (12%) patients, respectively. Comorbidities based on the Charlson index are summarized in Table 1. Medications that were the most frequently associated with ADRs were electrolytes or fluids (n = 623, 62%), followed by antibiotics (n = 569, 57%) and peptic ulcer drugs (n = 463, 46%) (Table 2).

**Table 1:** Characteristics and demographics of patients on admission

Characteristic	Mean ± SD or n (%) n = 962
Age (years)	70.0 ± 14.8
Male sex	517 (54)
Race (Japanese)	957 (99.5)
Admitting ward	
Medical	437 (45)
Surgical	410 (43)
Intensive care units	115 (12)
Comorbidity	
Myocardial infarction	67 (7)
Heart failure	141 (15)
Peripheral vascular disease	54 (6)
Cerebrovascular disease	136 (14)
Dementia	143 (15)
Chronic obstructive pulmonary disease	122 (13)
Rheumatologic	38 (4)
Peptic ulcer	247 (26)
Liver diseases	177 (18.4)
Diabetes	163 (16.9)
Chronic kidney disease	61 (6)
Any tumor	377 (39.2)

Most parameters are duplicated to a certain degree, as many patients experienced multiple medical events.

**Table 2:** Medications suspected to induce adverse drug reactions (ADRs)

Medication	n (%) n = 997
Electrolytes or fluids	623 (62)
Antibiotics	569 (57)
Peptic ulcer drugs	463 (46)
Sedatives	360 (36)
Antihypertensive	302 (30)
Laxatives	254 (25)
Diuretics	221 (22)
Cardiovascular	202 (20)
NSAIDs	194 (19)
Anticoagulants	170 (17)
Antidiabetics	139 (14)
Antipsychotics	119 (12)
Dyslipidemic agents	73 (7)
Analgesics	42 (4)

NSAIDs, nonsteroidal anti‐inflammatory drugs.

### 3.1 Distribution of NA score and percentage of ADRs by each component

NA components 6 through 10 (response to placebo if used, evidence in blood of toxicity, dose response, similar reactions before, and other objective evidence) classified more than 95% of suspected ADRs with a specific score; in which 99.8% (n = 1576) of suspected ADRs were classified with a score 0 (do not know) for component 6, and 99.9% of suspected ADRs were classified with a score 0 (no or do not know) for component 7. Thus, components 6 through 10 did not show sufficient categorization in identifying ADRs in this cohort. On the other hand, components 1 through 5 (previous conclusive report, time course, improvement after withdrawal or treatment, re‐emergence after re‐challenge, and other causative conditions of symptoms) showed good categorization in identifying ADRs from among suspected ADRs for each component; in which 64% (n = 1002) of suspected ADRs were classified with a + 1 score (yes) and 37% (n = 577) of suspected ADRs were classified with a 0 score (no or do not know) for component 1 (Table 3).

**Table 3:** Distribution of the Naranjo Algorithm (NA) score for each component

	Component	Score			
		+2	+1	0	–1
1	Are there previous conclusive reports on this reaction?	—	1002 (64)	577 (37)	—
2	Did the adverse event appear after the suspected drug was administered?	1172 (74)	—	400 (25)	7 (0.4)
3	Did the adverse reaction improve when the drug was discontinued or a specific antagonist was administered?	—	322 (20)	1257 (80)	—
4	Did the adverse reaction reappear when the drug was readministered?	309 (20)	—	1040 (66)	230 (15)
5	Are there alternative causes (other than the drug) that could on their own have caused the reaction?	761 (48)	—	422 (27)	396 (25)
6	Did the reaction reappear when a placebo was given?	—	3 (0.2)	1576 (99.8)	0 (0)
7	Was the drug detected in the blood (or other fluids) in concentrations known to be toxic?	—	2 (0.1)	1577 (99.9)	—
8	Was the reaction more severe when the dose was increased or less severe when the dose was decreased?	—	24 (2)	1555 (98)	—
9	Did the patient have a similar reaction on the same or similar drugs in any previous exposure?	—	35 (2)	1544 (98)	—
10	Was the adverse event confirmed by any objective evidence?	—	53 (3)	1526 (97)	—

Data expressed as n (%).

**Figure 2:**
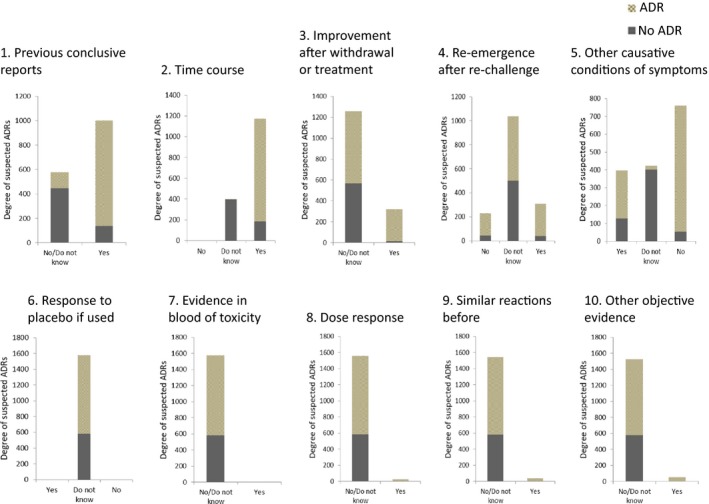
Distribution of adverse drug reactions (ADRs) by each Naranjo Algorithm (NA) component. The distribution of ADRs identified by physician reviewers for scored suspected ADRs by each NA component is shown. A total of 10 components, each consisting of 2 or 3 classifications were evaluated

Each NA component 1 to 5 had relatively high sensitivity or specificity for categorizing ADRs among suspected ADRs. With component 1, 86% (n = 866) of suspected ADRs were confirmed as ADRs among 1002 suspected ADRs assigned a + 1 score (Yes), and 23% (n = 131) of suspected ADRs were confirmed as ADRs among 577 suspected ADRs assigned a 0 score (No/Do not know) (Figure 2). Since the NA has a “Do not know” classification, we simply could not calculate specificity. When we classified “do not know” as “no”, the sensitivity was 0.87 and specificity was 0.77 for component 1. Similarly, the approximate sensitivity and specificity were 0.99 and 0.68, respectively, for component 2; 0.31 and 0.97, respectively, for component 3; 0.27 and 0.93, respectively, for component 4; and 0.71 and 0.91, respectively, for component 5.

### 3.2 Relationship between total NA score and ADRs percentage of suspected ADRs

**Figure 3:**
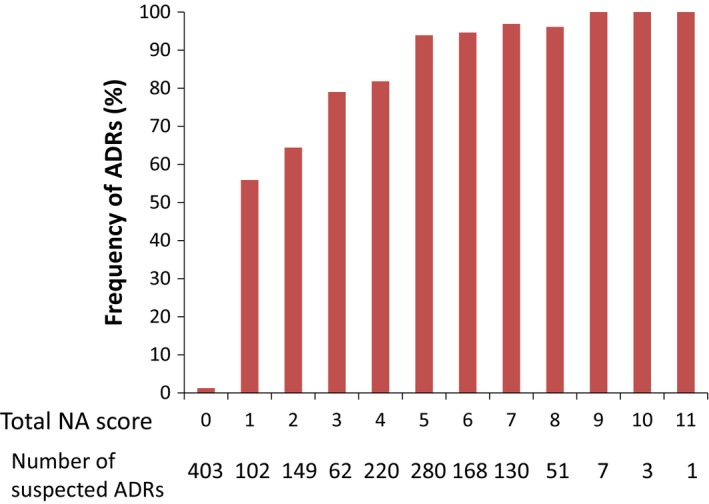
Relationship between the total Naranjo Algorithm (NA) score and the percentage of identified adverse drug events (ADRs) among suspected ADRs. The percentage of confirmed ADRs among suspected ADRs are expressed for each total NA score (0 through 11)

The total NA score calculated for each suspected ADR ranged from −2 to 11. The most frequent total NA score was 0 (n=403) followed by 5 (n=280). The percentage of ADRs was 56% if the total NA score was 1, and it gradually increased to 94% if the total NA score reached 5 (Figure 3). We did not show the total NA scores of −2 and −1 since only 2 and 0 suspected ADRs, respectively, were assigned these scores.

### 3.3 Sensitivity and specificity of the NA to determine ADRs

**Figure 4:**
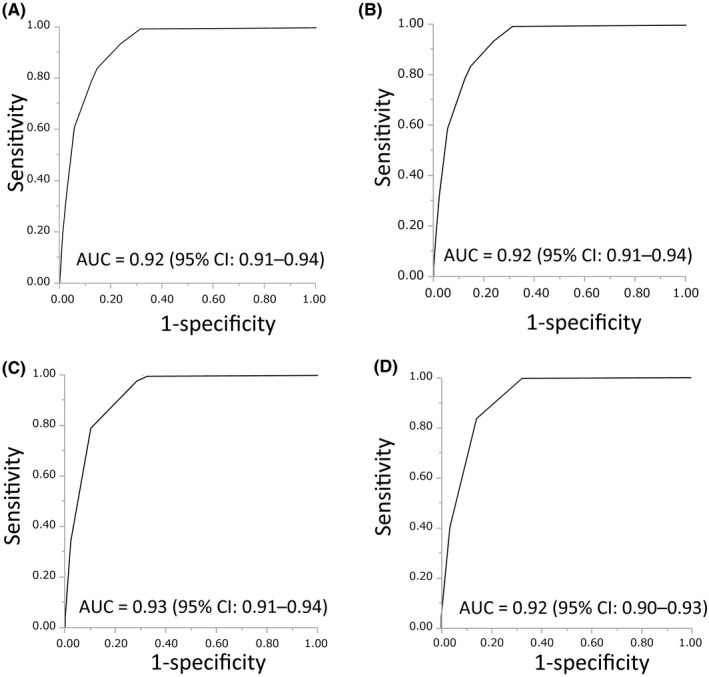
Receiver operating characteristic curve for adverse drug events (ADRs) and total Naranjo Algorithm (NA) score. A, The AUC for the sum of all NA components. B, The AUC for selected NA components (1‐5). C, The AUC for selected NA components (1‐5) converted to binary scores (0 or 1). D, The AUC for selected NA components (2‐5) converted to binary scores (0 or 1)

The area under the curve (AUC) to confirm ADRs was 0.92 (95% confidence interval [CI]: 0.91‐0.94) based on the total NA sore; the specificity was 0.94 and the sensitivity was 0.61 if the cut‐off value was set at 5 (Figure 4A). Since more than 97% of suspected ADRs were assigned a score of 0 for components 6 through 10, we considered that these components were not useful in the real‐world setting. We generated a modified NA that consisted of components 1 through 5. This modified NA confirmed ADRs with an AUC of 0.92 (95% CI: 0.91‐0.94), which was the same AUC as the original NA (Figure 4B). If the cut‐off value was set at 5, the specificity was 0.95 and sensitivity was 0.59. In the modified NA, we reclassified NA components 2, 4, and 5 into binary variables, which increased the specificity to 0.98 and sensitivity of 0.34 with an AUC of 0.93 (95% CI: 0.91‐0.94) if the cut‐off value was set at 4 (Figure 4C). We further modified the NA to consist of components 2 through 5 as binary variables. This simplest NA confirmed ADRs with an AUC of 0.92 (95% CI: 0.90‐0.93) and showed a specificity of 0.97 and sensitivity of 0.40 if the cut‐off value was set at 3 (Table 4, Figure 4D).

**Table 4:** Modified Naranjo Algorithm (NA)

	Component	Score	
		Yes	No/Do not know
2	Did the adverse event appear after the suspected drug was administered?	+1	0
3	Did the adverse reaction improve when the drug was discontinued or a specific antagonist was administered?	+1	0
4	Did the adverse reaction reappear when the drug was readministered?	+1	0
5	Are there alternative causes (other than the drug) that could on their own have caused the reaction?	0	+1

## 4 DISCUSSION

We showed that the NA was able to categorize ADRs among suspected ADRs efficiently in daily clinical practice using the large‐scale JADE database,^9^ which was independent with a consensus panel by physicians' reviewers. While each NA component showed relatively high sensitivity or specificity, we evaluated the sensitivity or specificity for the total NA score, since healthcare professionals usually make a decision from multiple factors in the actual clinical setting. We also showed that the modified NA, consisting of components 1 through 5, also effectively categorized ADRs with a high likelihood. We further modified the NA to include all binary scores for components 1 through 5 and found that this algorithm determined ADRs with high likelihood, also similar to the original. In addition, we removed component 1 because this component required sufficient knowledge of ADRs for each suspected drug. We considered that the modified NA with binary scores for components 2 through 5 was the most reasonable in terms of the practical use in daily clinical practice and its effectiveness in determining ADRs with a high likelihood, similar to the original index and all of the other modified NAs.

In previous studies, the NA was utilized retrospectively to evaluate the probabilities of ADRs in a specific case or cohort.^3^, ^4^, ^5^, ^6^ In this study, however, we showed that the NA had high predictive accuracy for determining true ADRs among suspected ADRs, which could contribute to safety monitoring activities by healthcare professionals or pharmaceutical manufacturers. If the modified NA score is simultaneously reported with a suspected ADR, a health authority or pharmaceutical manufacturers could evaluate the suspected ADR more easily and quickly and could allocate time and resources more effectively. For example, pharmaceutical manufacturers could start an intensive survey giving priority to a suspected ADR with a high modified NA score. Additionally, healthcare professionals could start preclinical studies to clarify the mechanism of ADRs focusing on a high modified NA score. Thus, the modified NA score could help healthcare professionals or pharmaceutical manufacturers take their own action in preventing ADRs as early as possible before health authorities issue a warning or guidance.

NA was reported to show poor performance for causality assessment of hepatic adverse reactions.^16^, ^17^ On the other hand, NA and modified NA were able to categorize ADRs among suspected ADRs including hepatic adverse reactions in the current study. However, the number of hepatic adverse reactions was limited in the current study, the reliability to assess such hepatic adverse reactions was uncertain. Further studies which address the accuracy of NA and modified NA against hepatic adverse reaction should be considered.

Other than the NA, Gallagher et al reported the usefulness of the Liverpool adverse drug reaction causality assessment tool.^7^ Although this tool also tried to simplify the NA and increase its credibility, their study had different objectives. It takes time to evaluate one case and provide an outcome (possible, probable, or definite) using the probability tree in the Liverpool tool. Additionally, this tool does not provide any score to be evaluated for sensitivity and specificity, similar to the NA. Also WHO‐UMC causality assessment could be another simple tool to categorize ADR.^18^ While this tool takes number of assessment criteria into consideration to categorize ADRs and each assessment criteria are similar to NA, it does not provide any score to be evaluated for sensitivity and specificity as well. Thus, there have been few reports proposing a tool that could be used to take action to mitigate adverseness and to prevent recurrence proactively rather than merely confirming the probability of ADRs retrospectively. We think our modified NA will not jeopardize the spontaneous ADR reporting but increase the awareness of ADR reporting with simple tool. It is still challenge for medical professionals to report suspected ADRs spontaneously because the importance of ADR reporting could not be understood well and medical professionals do not have an effective trigger tool to report ADRs. We are convinced that simple ADR assessment tools including our modified NA can introduce more frequent ADR reporting among medical professionals and can be used as a trigger tool to report ADRs.

Our study has several limitations. First, the JADE study only enrolled inpatients. Therefore, the modified NA score in this study might not be applicable in outpatients. Pharmacovigilance for inpatient should be different from usual pharmacovigilance situation of spontaneous reporting. Further studies are needed to clarify whether our findings could be applicable in outpatient settings and to generalize the modified NA for use in a pharmacovigilance system. Second, we removed components 6‐10 in the modified NA model. For drugs in which the blood level should be known, such as vancomycin or theophylline, component 7 could be useful for detecting ADRs. However, only 2 cases were given a score of +1 for that component in this study, which shows that measuring blood levels of suspected drugs is not frequent in daily clinical practice. Third, the same independent physician reviewer classified the ADR and scored NA at different times, which might have led to a connection between ADR classification and NA scoring and subsequently to misclassification of the NA based on the reviewer's background or knowledge. Fourth, the JADE study only enrolled Japanese patients. To generalize the results globally, we need to study the modified NA in other countries to evaluate its ability to categorize ADRs among various races and in different healthcare systems, which affect decision‐making by healthcare professionals. Fifth, the JADE study was conducted in 2004 and the data used seemed relatively old. However, NA was developed in 1981 and still used for clinical settings. The drug used in this study and spontaneous ADR reporting system has not been changed for decades. Thus, the findings and clinical implication of this study should be valid at present time. Finally, we focused on the most suspected drug among all drugs administered when symptoms occurred in this study. Therefore, we could not exclude the possibility of synergistic effects of multiple drugs and drug‐drug interaction.

In conclusion, we assessed the categorization abilities of the original and modified NAs in daily practice and found that the modified NA could be easily used to categorize actual ADRs among suspected ADRs with high predictive accuracy. Therefore, use of the modified NA could help to save time and resources and categorize ADRs more effectively and promptly in daily clinical practice. Additionally, utilizing this tool for a pharmacovigilance system could be useful to enable professionals take prompt action in developing a strategy to prevent and mitigate the adverseness of ADRs.

## DISCLOSURES

H. Murayama and Y. Takahashi: Employees of Novartis Pharma KK; M. Sakuma and T. Morimoto: None Declared.
